# Action for better brain health among people living with HIV: protocol for a randomized controlled trial

**DOI:** 10.1186/s12879-021-06540-7

**Published:** 2021-08-20

**Authors:** Adria Quigley, Marie-Josée Brouillette, Lesley K. Fellows, Nancy Mayo

**Affiliations:** 1 Centre for Outcome Research and Evaluation (CORE), McGill University Health Centre (MUHC), 5252 de Maisonneuve, Montreal, QC H4A 3S5 Canada; 2 Department of Psychiatry, McGill University, 1033 Pine Avenue West, Montreal, QC H3A 1A1 Canada; 3 Department of Neurology and Neurosurgery, McGill University, 3801 University St, Montreal, QC H3A 2B4 Canada; 4 Department of Medicine, McGill University, 1001 Decarie Boulevard, Montreal, QC H4A 3J1 Canada; 5 School of Physical and Occupational Therapy, McGill University, 3654 prom Sir-William-Osler, Montreal, QC H3G 1Y5 Canada

**Keywords:** Goal management training, Cognitive rehabilitation, Physical activity, Healthy lifestyle, Brain health, HIV

## Abstract

**Background:**

Lifestyle changes can protect or improve brain health in older adults. However, sustained lifestyle change is difficult for everyone and may be more difficult for those with executive dysfunction, including some people living with HIV. Thus, the key question is how we can improve adherence to the most promising interventions among people living with HIV experiencing cognitive difficulties. Goal management training is a cognitive rehabilitation program that targets executive dysfunction by teaching goal-directed behaviour and self-management. It is a promising means to improve adherence to lifestyle interventions.

**Objective:**

To estimate the extent to which goal management training before a healthy lifestyle program is associated with greater adherence to health recommendations, achievement of health-related goals, and better brain health and health outcomes compared to the healthy lifestyle program alone among people living with HIV.

**Methods:**

Brain Health Now cohort participants with cognitive difficulties or are not aging successfully are eligible. All participants will be given health resources, a health coach, a goal-setting digital application, and access to an online goal-setting workshop. The intervention group will participate in nine 2-h goal management training sessions and then will enter the healthy lifestyle program. Control participants will enter the healthy lifestyle program directly. A total sample of 100 participants will participate for 12 months. The main outcome is adherence to the healthy lifestyle program, defined as the number of weeks where physical activity adherence targets were met (150 min per week, measured with an activity monitor). Weekly social activities will be captured via self-report with confidential photo validation. We will send weekly health state reports to the participants. Downstream outcomes include cognitive ability, health-related quality of life, mobility, vascular risk profile, and social network size. We will analyze the data using a linear regression model.

**Discussion:**

This project is the first to test whether goal management training can augment adherence to health recommendations among individuals with cognitive difficulties. If successful, behavioural interventions such as goal management training could be implemented as an adjunct to lifestyle interventions in other clinical populations.

*Trial registration:* This trial was registered on clinicaltrials.gov (NCT04345484) on April 14, 2020, https://clinicaltrials.gov/ct2/show/NCT04345484?term=NCT04345484&draw=2&rank=1.

## Background

### Successful aging with HIV

Successful aging has become a priority among people living with HIV as they live longer with this chronic condition. There are three proposed components of successful aging: high cognitive and physical functioning, low levels of disease and disability, and active engagement in life [1]. It is estimated that 30–60% of people living with HIV experience cognitive difficulties [2–4]; however, these estimates are susceptible to selection bias and may overestimate the prevalence of cognitive difficulties [5]. Given the proportion of people living with HIV with poor brain health, strategies are needed to help these individuals age successfully.

### Lifestyle interventions

High quality evidence from HIV-negative individuals shows that healthy lifestyle changes including physical activity [6–11], diet [12], social activity [13], and cognitive training [14] can protect or improve brain health. There is also evidence that lifestyle change can improve cognitive ability in people living with HIV [15–20]. A cross-sectional study of 139 people living with HIV determined that an increasing number of active lifestyle factors (physical activity, social activity, and employment) was associated with 41% lower prevalence of HIV-associated neurocognitive impairment [17]. Furthermore, the investigators determined that each of the lifestyle factors (physical activity, Cohen’s d = 0.58; social activity, Cohen’s d = 0.41; employment, Cohen’s d = 0.57) was associated with better cognition.

Despite the evidence that lifestyle factors are associated with better cognitive ability, only 51% of people living with HIV are meeting the World Health Organization physical activity recommendations, compared to 65% of HIV-negative individuals [21]. Relative to other chronic conditions, people living with HIV are among the most physically inactive, taking only 5899 steps per day on average, according to a meta-analysis of 24 studies [22]. Three meta-analyses [23–25] have reported variable adherence (61–100%) and high withdrawal rates (20–29%) among people living with HIV who participate in aerobic and resistance exercise trials. Sustained lifestyle change is difficult for everyone, but may be harder for those with executive dysfunction, including many people living with HIV [26]. Thus, the key question is how we can improve adherence to the most promising interventions in people living with HIV experiencing cognitive difficulties.

Goal management training (GMT) has become a recommended cognitive rehabilitation strategy to improve executive function among older adults [27]. GMT is a standardized program delivered over 9 weeks in small groups that targets executive dysfunction by teaching goal-directed behaviour, self-management, and mindfulness [28]. GMT also trains participants to use explicit strategies to reduce cognitive load in everyday tasks, and methods to cue attention to maintain focus on specific tasks, through weekly 2 h sessions and home practice. A systematic review and meta-analysis of 15 studies determined that GMT had a small to moderate effect on executive function immediately following the intervention (Hedges’ *g* = 0.227) and at follow-up (Hedges’ *g* = 0.549) among healthy individuals and those with conditions affecting cognitive performance [27].

GMT has been tested very little among people living with HIV. A study of 90 people living with HIV who were randomized to a single session of GMT, GMT plus metacognitive training, or control (education and origami task) found moderate effects of the combined GMT groups on everyday multitasking performance and metacognitive tasks compared to controls [29]. GMT has not been used to augment goal-directed lifestyle changes among people living with HIV.

This study will test an innovative approach to enhance adherence to lifestyle interventions, building on feasibility evidence from pilot trials in the Brain Health Now cohort study, where we have tested three brain-health-promoting interventions, including computerized cognitive training, GMT, and exercise. Feasibility was demonstrated for both GMT and exercise, based on the success of recruitment and retention, improvements in brain health outcomes, and participant feedback [30, 31]. The varied adherence observed in these pilot trials suggests that people living with HIV who have cognitive difficulties may need more assistance to make and sustain behaviour changes. While GMT was developed to improve goal-directed behaviour in general, we recognized that this cognitive rehabilitation approach could be applied to improve the ability to achieve health-promoting lifestyle goals in particular. We therefore hypothesize that GMT priming before a personalized healthy lifestyle program (HLP) will improve adherence to the program and subsequently improve health outcomes, compared to the HLP without GMT priming in people living with HIV experiencing cognitive difficulties.

### Study objectives

The overall objective of Action for Brain Health Now is to understand, empower, and act to protect and improve brain health in HIV, and equip patients to take charge of their brain health. This sub-study is directed to the third aim, act. This proposed study addresses the real-world challenge of implementing tailored recommendations, recognizing that individuals with cognitive difficulties who would benefit the most from lifestyle changes are less equipped to successfully adopt and sustain healthy behaviours. We will trial a novel cognitive rehabilitation approach using GMT to boost adherence to a tailored active living intervention.

### Specific objective

To estimate the extent to which goal management training before a personalized healthy lifestyle program is associated with greater uptake of health recommendations, achievement of health-related goals, and better brain health and general health outcomes compared to the healthy lifestyle program alone.

## Methods

### Design

This is a single-blind, language stratified, parallel group randomized controlled trial. This trial was registered on clinicaltrials.gov (NCT04345484). This protocol is informed by the Standard Protocol Items: Recommendations for Interventional Trials (SPIRIT) checklist. This study is ongoing.

### Population

This is a sub-study of the Action for Brain Health Now (ABHN) cohort (http://brainhealthnow.mcgill.ca), which consists of 840 people living with HIV [32–34]. The extension for ABHN will involve 4 yearly visits for all participants. This will provide longitudinal data on the evolution of brain health trajectories in HIV over a minimum of 3 years. More than 20% of the cohort will be over 70 by the end of this study.

Any member of the ABHN from four existing sites (Special Immunology Services Clinic in Hamilton, Maple Leaf Clinic in Toronto, Clinique Médicale l’Actuel in Montreal and the McGill University Health Centre in Montreal) who is willing to undertake the intervention activities is eligible for the trial, provided they: Have one or more indicators of cognitive difficulties [35], defined here as a Brief Cognitive Ability Measure (B-CAM) [36] score or Communicating Cognitive Concerns Questionnaire (C3Q) [37] score (or both) below cohort median values (56/100 for B-CAM, 27/36 for C3Q) or do not meet successful aging criteria, with successful aging defined as meeting or exceeding Canadian norms on 7 or more of 8 health-related quality of life domains on the RAND-36 [38]. Participants will be excluded if they previously participated in GMT.

For new participants entering the cohort, the eligibility criteria are: Age ≥ 45; HIV + for at least 1 year; ability to communicate adequately in either French or English; and ability to give written informed consent. The exclusion criteria for new participants are as follows: Dementia (Memorial Sloan Kettering-rating stage 3 or more on the cognitive component only); life expectancy less than 3 years or other personal factor limiting the ability to participate in follow-up; non-HIV-related neurological disorder likely to affect cognition; known active central nervous system opportunistic infection or hepatitis C requiring interferon treatment during the follow-up period; known psychotic disorder; current substance dependence or abuse within the past 12 months; or previous participation in GMT.

### Recruitment

Our team will do a search among our cohort participants and create a list of potential participants. If these potential participants have agreed in the main study consent form to be contacted for sub-studies, they will get a phone call, email, or will be recruited during their regular study visit by the research coordinator involved in the main study. If cohort participants decline to participate in this sub-study, we will ask them their reasons for declining [5]. Those who agree to participate in the study will sign the consent form provided by the research coordinator. We have received conditional approval from the McGill University Health Centre Research Ethics Board (ABHN_Goals 2020-6202).

### Intervention

**Fig. 1:**
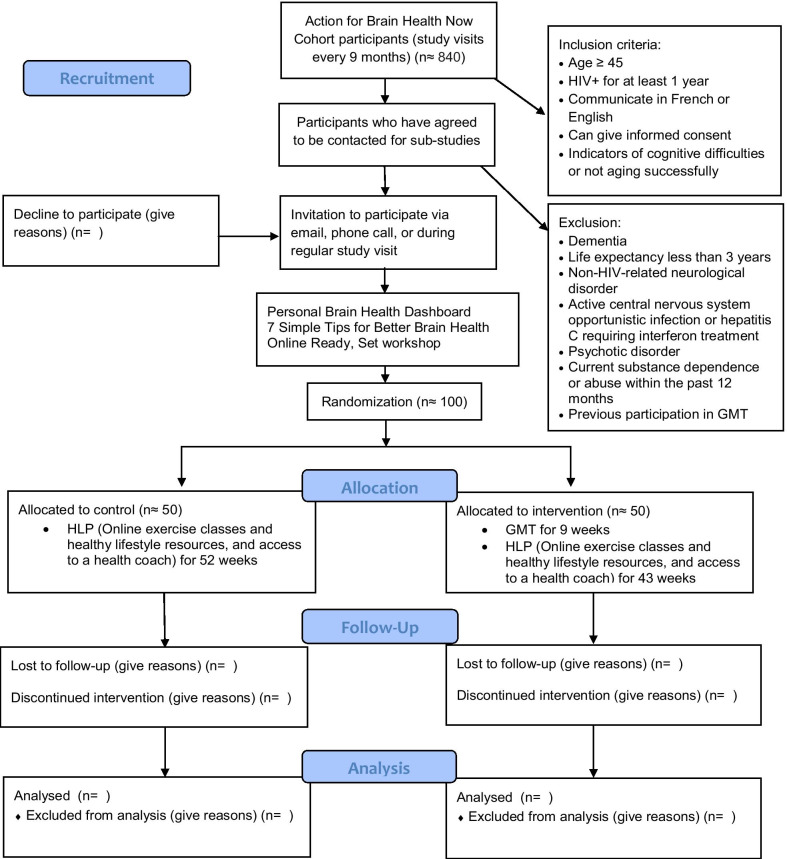
Participant flow diagram

The flow of participants through the intervention trial is shown in Fig. 1.

*Ready*: All study procedures will occur online. All cohort participants will receive a personalized *My Personal Brain Health Dashboard* populated directly from their study data, which provides them with information on 15 areas including cognitive ability, health outcomes, health-related quality of life, and lifestyle factors. Participants also receive a document with *7 Simple Tips for Better Brain Health*. In addition, the participants of this trial will receive a list of relevant local resources (e.g., community activities and groups, online exercise classes, smoking cessation programs). We offer this to the cohort participants, as it represents current best practices for health promotion: personalized risk assessment, tailored recommendations, and concrete suggestions for action [39]. However, this generic approach may not address the needs of those with brain health challenges.

All of the participants who agree to enter the trial will be invited to attend the online Ready, Set Workshop which will occur before randomization and will last approximately four hours. As part of the workshop, all of the participants will be shown via pre-recorded videos how to interpret the *My Personal Brain Health Dashboard*, use their activity tracker, and use two digital applications (a goal-setting app and the MyHealth app). Participants will be sent the Garmin vívofit 4 Activity Tracker via Canada Post. Participants who do not have an app compatible device such as a smartphone or tablet will be provided with a smartphone for the duration of the study.

*Set*: One of the short video modules will include instruction on specific, measurable, attainable, relevant, and time-bound (SMART) goals and demonstration of the goal-setting app to use for recording and monitoring goals. The app will guide all the participants through the steps to write and follow up on their SMART goals. Participants will be encouraged to be as specific as possible when filling in a box on the app with an aspect of their health that they wish to improve. The goal-setting app will give them feedback on their goals through simplified Goal Attainment Scaling [40]. Participants will have the option to contact other participants with similar goals via their health coach. For example, if multiple participants have goals related to yoga, the coach can suggest they communicate and help each other achieve their goals, as long as the participants have consented to this option.

*Go*: Following the workshop, the participants will be randomized. Randomization will be done in a 1:1 ratio by the study statistician using randomization.com. Stratification by language will be carried out, as GMT will be done in either French of English. The intervention group will participate in GMT for 9 weeks and then will enter the HLP for 43 weeks. Control participants will enter the HLP directly and will participate for 52 weeks. It is not possible to blind participants in this type of study.

### Healthy lifestyle program

The HLP includes access to a list of free online resources, a health coach, and physical activity monitoring (step count, time spent in various cadence bands, and sedentary time) using the Garmin vívofit 4 activity tracker and recording and monitoring of goals with the goal-setting app. Considering that gyms are high risk in the COVID-19 era, we will provide participants with links to online exercise and yoga classes, which they can do in their home with minimal equipment. We have also created two 30–40-min bootcamp classes. Participants will be encouraged to follow the bootcamp class twice a week and take a yoga class once a week. Once they are comfortable, participants will be encouraged to take other online fitness classes. Those who have a medical contraindication to moderate exercise (chronic heart failure, angina, history of myocardial infarction) will be recommended to use only the yoga resources for exercise. All participants will be assigned a health coach to facilitate the transition into using community resources to meet their goals. The health coach will provide feedback, encouragement, and suggestions for increasing activity via weekly interactions throughout the HLP.

### Goal management training

The intervention group will participate in GMT, a standardized cognitive rehabilitation program, which will be provided per the manual. It consists of 9 online sessions, once per week, for two hours [41]. Participants will be given homework to complete between the sessions. There are five steps in GMT [28]. In stage 1, participants are asked to stop and ask what task they are trying to accomplish. They select their goals in stage 2, and these goals are partitioned into smaller steps in stage 3. Stage 4 involves encoding and retention of goals and steps. In stage 5, participants monitor their outcomes to make sure they align with their original goals [28].

### Outcome measures

Refer to Table 1 for a list of outcome measures that will be administered in this study.

**Table 1:** Outcome measures

Outcome	Measure	Value
*Confirmatory outcomes*		
Primary 1: uptake of the recommendations for physical activity	Number of physical activity-weeks. Recorded by Garmin VivoFit 4	Number of weeks in which physical activity guidelines are met (150 min of moderate to vigorous activity in bouts of 10 min) [42]
Primary 2: uptake of the recommendations for social activity	Number of social activity-weeks. Recorded by pictures provided by participants	Number of weeks in which at least one social activity was attended
*Explanatory outcomes*		
Achievement of personal goals	Goal attainment scaling [40, 43] Recorded on the goal-setting app	N and diversity of goals set and met
Physical activity pattern	Daily step counts, time spent in cadence bands for moderate (100 steps/min) and vigorous (130 steps/min) intensity [44], sitting time. Recorded by the Garmin VivoFit 4	Trajectory of weekly cumulative step counts (rising, stable, falling) Trajectory of weekly cumulative sitting time (rising, stable, falling)
General Health perception, sleep, distress, pain, fatigue, depression, anxiety, quality of life	Visual Analogue Health States [45]	0–10 for each
*Downstream health outcomes (platform measures)*		
Mood	RAND-36 Mental Health Inventory [46]	0–100
Energy/fatigue	RAND-36 Vitality [46]	0–100
Physical function	RAND-36 Physical Function Inventory [46] and five-repetition sit-to-stand test [47]	0–100; time in seconds
Performance based-report cognitive ability	Brief Cognitive Ability Measure (B-CAM) [48]	0–35 English / 42 French
Self-report cognitive ability	Communicating Cognitive Concerns Questionnaire (C3Q) [37]	0–36
Health related quality of life	EuroQol Five Dimensions Questionnaire (EQ-5D) [49], Patient Generated Index [50, 51]	0–1; 0–100
Improved vascular risk profile	Framingham Cardiovascular Disease Risk Score [52, 53]	Probability of stroke
Social network size	Social network size and quality [54]	0–100

### Confirmatory outcomes

The main confirmatory outcome will be physical activity and social activity adherence to the HLP, hypothesized to be greater with GMT priming. The former will be measured by counting the number of weeks that physical activity adherence targets were met, i.e. 150 min per week of moderate to vigorous exercise, accumulated in bouts of at least 10 min [42]. This outcome will be assessed continuously with the activity monitor (*Garmin VivoFit 4)*. The latter adherence outcome will be the number of documented social activities per week captured through self-report with confidential photo validation. For example, if they attend a yoga class or a painting class, they can take a picture of the yoga studio or art studio and send to a secure email address. A research team member will record the details of the activity and then the picture will be deleted. Participants will be instructed to not take pictures of other people and are not obliged to appear in the picture.

### Explanatory outcomes

Principles of Goal Attainment Scaling [40, 43] will be used to assist with goal-setting. Participants will use the digital goal-setting application developed by our team to set at least three goals, actions for each goal, how difficult the goal will be to achieve, and the importance of the goal. Weekly and monthly reminders will be sent to participants to determine their progress in achieving their goals.

Physical activity patterns including daily step counts, time spent in cadence bands for moderate (100 steps/min) and vigorous (130 steps/min) intensity [44], and sitting time will be recorded using the Garmin VivoFit 4 activity tracker. Garmin activity trackers have been widely used to measure step count, physical activity, and sedentary time and have established validity and reliability [55].

**Fig. 2:**
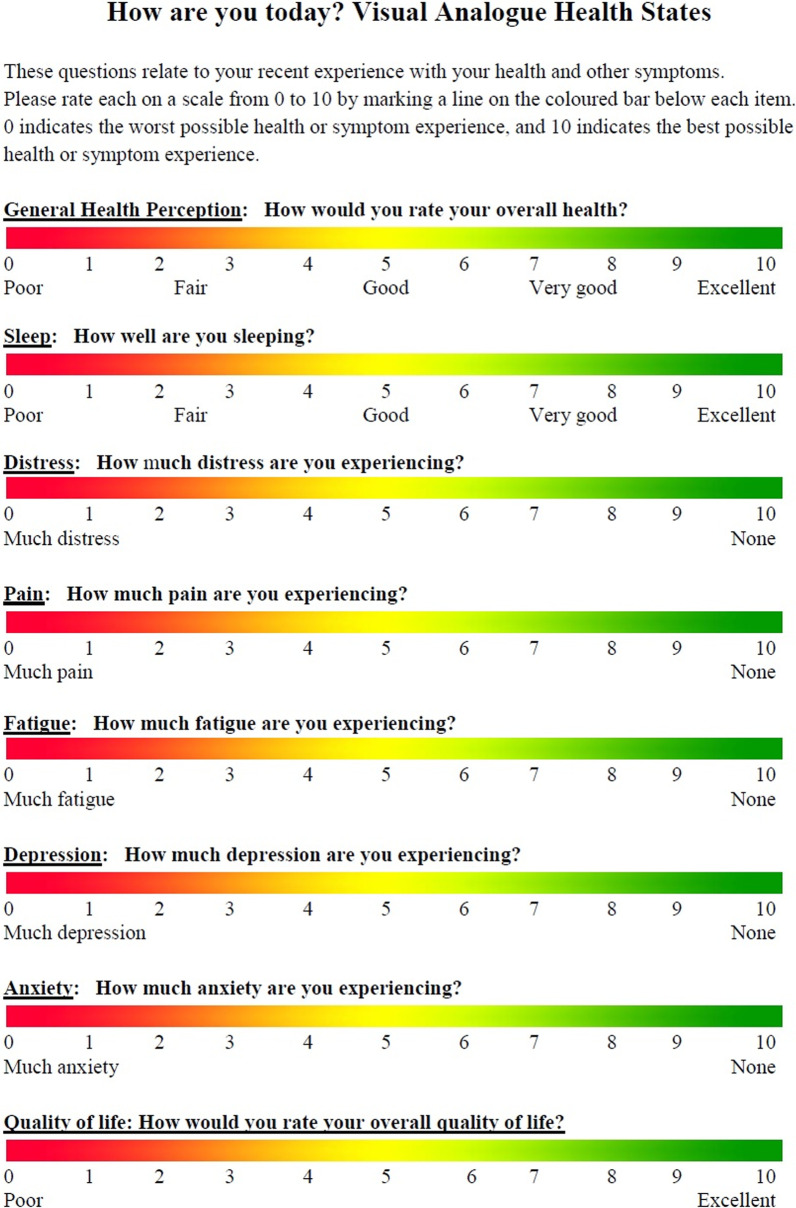
Visual analog health states questionnaires

The MyHealth app will be used to send visual analog scale health states questionnaires to the participants. A questionnaire will be sent every week and the participants will have the 48 h to respond to it. There are 8 questions about their level of pain, anxiety, depression, sleep, distress, fatigue and health perception using the validated Visual Analog Scale [45]. See Fig. 2 to view the questionnaire.

### Downstream measures

The downstream measures will be administered during their regular ABHN study assessments by a blinded trained research coordinator. The ABHN visits are scheduled every 9 months so all of the participants in the trial will have at least one visit during the period of the trial and one after the trial is completed. Due to COVID-19 restrictions, these assessments are conducted virtually.

The EuroQol Five Dimensions Questionnaire (EQ-5D) measures five aspects of health-related quality of life, including mobility, self-care, usual activities, pain and discomfort, anxiety and depression. There is evidence of construct and convergent validity, as well as responsiveness to treatment and changes in health status [56]. The physical functioning (10 items), mental health (5 items), and vitality (4 items) subscales of the RAND-36 will be used to measure aspects of health-related quality of life [46]. The RAND-36 has good to acceptable internal consistency, construct validity, and responsiveness [56]. It has been used widely in the literature [57]. Physical function will also be assessed using the five-repetition sit to stand test, which has been used to predict fall risk among people living with HIV [58].

We will measure cognitive ability using the Brief Cognitive Ability Measure (B-CAM), a brief computerized cognitive test developed by our team using Rasch analysis where higher scores reflect better cognitive ability [32, 59]. The B-CAM includes tests of reaction time, response inhibition [60], memory and learning, working memory [61], visuospatial memory [62], verbal fluency, and executive function [63, 64]. Self-reported cognition will be assessed using the Communicating Cognitive Concerns Questionnaire (C3Q), an 18-item questionnaire that estimates the frequency of cognitive difficulties among people living with HIV on a three-point scale with higher scores reflecting less frequent cognitive difficulties [37, 65].

### Data collection

The data of participants included in the study will be stored in a secure server held at the McGill University Health Centre Research Institute, accessible only to the research team. The data will be stored in conjunction with the participants’ anonymous codes. Any participant’s identification will take place through a separate information source and external to the system, making it possible for the research team to reconstruct the correspondence between the anonymized data and the participants enrolled in the study. Data backup will be made daily and automatically in order to properly secure data storage. The principal investigator will assure the quality of computerized data for this study. Those collecting and analyzing the data will be kept blind to group assignment.

### Ethical considerations

All procedures will be in accordance with the Helsinki Declaration. Any amendments to the study protocol will be submitted for review to the research ethics board. Adverse events will be immediately reported to the research ethics board and the participants will be directed to obtain treatment if needed. Protocol violations or deviations will be reported to the principal investigator. Protocol exemptions, violations, and deviations will be logged. All participant-related information including the questionnaires, evaluation forms, and reports will be kept strictly confidential. All records will be kept in a secure, locked location and only research staff will have access to the records. Participants will be identified only by means of a coded number specific to each participant, and a participant letter code. All computerized databases will identify participants by numeric codes only and will be password protected. Participants will consent to have their MyHealth app data stored with DataRiver in the informed consent form prior to the collection of any research data. DataRiver will destroy all the data and wipe hard drives clean and the storage solution as well, once the data has been transferred to the principal investigator and the study is closed. Upon request, clinical information may be reviewed by or released to auditors, funders, or regulatory agencies. Participants will be given the option to withdraw from the study at any time with no effect on their care or ability to remain in the cohort study.

### Statistical analysis

The analysis assumes that the distribution of physical activity-weeks at the end of the study period will be near normal, permitting a linear regression model; if not, a Poisson model can be used. Both intention-to-treat, with imputation and sensitivity analyses, and per protocol analyses will be conducted. Intention-to-treat will address the benefit of offering GMT as a service to all with need, while the per protocol analysis will address the benefit to those who are able to adhere to the program and will help identify characteristics (including sex and gender) of those most able to benefit. We will follow the Sex and Gender Equity in Research (SAGER) [66] guidelines and will present study data disaggregated by sex and gender. To address bias resulting from missing data, we will conduct multiple imputation on the data for outcomes with sufficient data [67].

### Sample size

Sample size calculations were based on estimates of usual rates of adherence to physical activity for Canadian adults estimated at 10–20% of the time [68], translating to approximately 9 of 52 weeks for the control group and a desired 22 weeks (50% of 43 weeks) in the GMT priming group, which is the targeted adherence rate for interventions for cardiovascular risk factor improvement [69]. As we expect that elements of the HLP program will lead to some activity improvement in the control group (+ 4 weeks), we will power our study to detect a minimum difference of 9 activity weeks between groups over 43 weeks with an estimated SD of 11 weeks. With adjustment of the variance (standard deviation) by 15% to account for dropouts and the need to impute data, this yields an effect size of 0.71 [70]. For 90% power and risk of Type I error of 0.05, the required number of people is 38 participants per group. We will therefore target a total sample of 100 (50 per group) to account for uncertainty in our sample size assumptions and incomplete data. This sample size will also be sufficient to permit other statistical models to be used in the case of non-normality, such as ordinal regression.

Taking advantage of the trial being embedded in the cohort, we will also compare platform outcomes for trial participants with platform outcomes for people who were eligible for the trial but not enrolled, including those at other sites, providing information about how the HLP with or without GMT compares to tailored brain health risk recommendations alone.

### Knowledge translation

Realize and the Canada-International HIV and Rehabilitation Research Collaborative (CIHRRC) will play key roles in disseminating study results to people living with HIV, policy makers, and health professionals through their websites, social media, e-modules and interprofessional learning curricula. Study methods and results will be presented either in poster or podium format at national and international conferences, such as the Canadian Association for HIV Research and the International Workshop on HIV and Aging. A peer-reviewed article will be submitted to a scientific journal for dissemination of the results.

## Discussion

HIV is a chronic condition that affects cognitive ability, with further negative effects from age and the accumulation of comorbidities. Strategies are needed to help these individuals self-manage the sequelae of this condition. People living with HIV have high rates of physical inactivity and poor aerobic fitness [22, 71, 72]. Dropout rates from physical activity interventions are higher among people living with HIV than in many other chronic conditions [25]. This project will be the first to test whether GMT can augment adherence to health recommendations among people living with HIV experiencing cognitive difficulties. This work aligns with three research priorities outlined by CIHRRC, including exploring community and social participation aging with HIV; identifying strategies for chronic disease management and healthy aging with HIV and; determining the effectiveness of rehabilitation interventions to support healthy aging with HIV [73].

Strengths of our approach include drawing participants from the ABHN cohort, with strong assessment protocols and outcome measures developed by our team. This project has shifted to a virtual format, which will mean a reduced time commitment and fewer environmental barriers to participation. This project offers real-world application as participants will have access to a personalized healthy lifestyle program. Other important features of this program include giving participants many healthy lifestyle activity choices along with support from a health coach and resources to incorporate self-management strategies. This project is not immune to challenges. Activity trackers are known to underestimate non-aerobic types of physical activity such as resistance training and yoga [74]. To address this challenge, the goal-setting app will track participants’ ability to achieve physical activity goals.

People living with HIV, clinicians, researchers, and policymakers are searching for strategies to improve adherence to lifestyle interventions. This study can contribute pragmatic evidence for the feasibility, effectiveness, and acceptability of a GMT intervention for people living with HIV experiencing cognitive difficulties. If this intervention is deemed successful, behavioural interventions such as GMT could be implemented as an adjunct to lifestyle interventions for people with cognitive difficulties in other clinical populations.

## Data Availability

Upon request, clinical information may be reviewed by or released to auditors, funders, or regulatory agencies.

## Abbreviations

ABHN: Action for Brain Health Now
B-CAM: Brief Cognitive Ability Measure
C3Q: Communicating Cognitive Concerns Questionnaire
CIHRRC: Canada-International HIV and Rehabilitation Research Collaborative
GMT: Goal management training
HLP: Healthy lifestyle program
SAGER: Sex and Gender Equity in Research
SPIRIT: Standard Protocol Items: Recommendations for Interventional Trials (SPIRIT)
